# A Comparative Study to evaluate Parent’s Ability to assess Dental Fear in their 6- to 10-year-old Children using Children’s Fear Survey Schedule—Dental Subscale

**DOI:** 10.5005/jp-journals-10005-1512

**Published:** 2018-06-01

**Authors:** Ritika Malhotra, Kapil Gandhi, Dipanshu Kumar, Shilpa Ahuja, Rishabh Kapoor, Anchal Sahni

**Affiliations:** 1 Reader, Department of Pedodontics, Inderprastha Dental College & Hospital, Ghaziabad, Uttar Pradesh, India; 2 Professor and Head, Department of Pedodontics, Inderprastha Dental College & Hospital, Ghaziabad, Uttar Pradesh, India; 3 Reader, Department of Pedodontics, Inderprastha Dental College & Hospital, Ghaziabad, Uttar Pradesh, India; 4 Senior Lecturer, Department of Pedodontics, Inderprastha Dental College & Hospital, Ghaziabad, Uttar Pradesh, India; 5 Senior Lecturer, Department of Pedodontics, Inderprastha Dental College & Hospital, Ghaziabad, Uttar Pradesh, India; 6 Postgraduate Student, Department of Pedodontics, Inderprastha Dental College & Hospital, Ghaziabad, Uttar Pradesh, India

**Keywords:** Anxiety, Children’s Fear Survey Schedule—Dental Subscale, Dental fear.

## Abstract

**Aim:**

Parental presence often provides unique challenge in dental operatory and is directly related to the quality dental treatment. The present study was done to investigate parent’s ability to assess dental anxiety of their 6- to 10-year-old child and to determine how parent’s and children’s fear assessments correlate with each other.

**Materials and methods:**

Prior to dental treatment, 94 child-parent combinations were included to complete Children’s Fear Survey Schedule—Dental Subscale (CFSS-DS) questionnaire, and Frankl score was assigned to children during treatment by operator.

**Results:**

Mean dental anxiety score reported by the children was 27.74, whereas by their parents was 39.64. There was a poor consistency of parents to predict their child dental fear (p < 0.05). Parents reported higher dental fear for their children.

**Conclusion:**

Parents assessment of their child’s fear may vary in accordance to factors, including their own dental fear. Such input may prevent dentists from establishing an accurate association with the child’s patient.

**How to cite this article:** Malhotra R, Gandhi K, Kumar D, Ahuja S, Kapoor R, Sahni A. A Comparative Study to evaluate Parent’s Ability to assess Dental Fear in their 6- to 10-year-old Children using Children’s Fear Survey Schedule—Dental Subscale. Int J Clin Pediatr Dent 2018;11(3):205-209.

## INTRODUCTION

Anxiety and fear of dental treatment in child patients have been recognized as a possible taxing entity in patient management. Dental fear and anxiety (DFA) are common among children, adolescents, and adults of all cultures. It has been observed that identification of dental fear may lead to dental neglect, which is a problem for both dentist as well as patient. Early recognition and management of dental fear is a key to an efficacious treatment for a child patient. Subsequently, there are four types of measurements that have been used for evaluating the dental fear in children: Behavior rating scales during dental visits (e.g., Frankl’s scale),^1^ physiological measures (e.g., pulse rate, blood pressure, muscle tension etc.),^2^ projective techniques (e.g., children’s dental fear picture test),^3,4^ and various other psychometric scales. Among these psychometric methods, CFSS is a fear scale for young children. Children’s fear survey schedule along with its dental subscale has been shown to be better than other scales, such as Venham’s picture test^3^ and dental anxiety scale.

The combined prevalence of DFA from 12 populations in Australia, Canada, Europe, and the USA was 9.4%. A study of 2,144 Dutch children, aged between 4 and 11 years, reported that girls were more fearful than boys, whereas 8- to 9-year-old were more fearful than other age groups.^6^ Anticipation of dental fear in children by parents may lead to dental neglect, which has a problem for both dentists and patients. Many patients are referred to pediatric dentists due to dental management behavioral problems (DMBP) emanating from dental fear. Careful assessment of dental fear in children or adolescents enables a customized approach to behavioral management and treatment of DFA, which correlate positively with a child’s behavior during dental treatment. The more fearful a patient is, the more behavioral problems can be expected. In a study of 3,204 urban Swedish children aged between 4 and 6 and 9 and 11 years showed that 61% of children with DFA had DMBP, but only 27% of children with DMBP had DFA as it may be due to any general fears, maternal dental fear, or age.^7^ Previous parental dental anxiety and their experiences may lead to interfere in child’s behavior during dental treatment. There are various theories regarding the philosophy of dental fear. A child’s dental fear can be associated with anticipation of dental treatment and frequent dental visits. Any aggressive dental treatment during younger age group in a child’s dental history plays a significant role in the procurement of dental fear. It is also observed that child’s temperament, cultural background, and subjective experiences are other important factors responsible for dental fear.^8^

The aim of the present study was to evaluate whether parents are able to accurately assess their child’s dental fear and patient behavior during dental treatment.

## MATERIALS AND METHODS

### Sample Collection

The present study was carried out in the Department of Pedodontics and Preventive Dentistry in Inderprastha Dental College & Hospital, Ghaziabad. Samples of 94 child-parent combinations with children 6 to 10 years, with or without previous dental experiences or referred by general dentist due to dental fear were included in the study. Patients with an eloquent intellectual disability, any physical restriction, psychiatric illness, and those who were accompanied by an adult other than a parent were excluded. If a patient met the criteria, his/her parent was given an explanation of the study and was invited to participate. If both concurred, assent and consent for the study were attained following completion of patient check-in procedures and before dental treatment.

### Questionnaire

Children’s fear survey schedule is used as assessing tool to predict the dental fear. It includes two interpretations: A self-rendition by the child and parent version.^9^

In the present study, CFSS-DS, a fear scale for young children, was used that was designed by Scherer and Nakamura^9^ and later revised to include specific dental fear items as one of its subscales by Cuthbert and Melamed.^10^ To overcome the language problem, this scale was also translated into Hindi, and therefore both English and Hindi versions were used.

This scale includes a set of 15 questions. The response ranges in 5-point scale, i.e., 1—not afraid at all; 2—very less; 3—moderate fear; 4—pretty much afraid; 5—very much afraid, with a score range from 15 to 75. Score of 38 and above indicates high dental fear.

### Study Design

Once avocation of child-parent dyad was done, full CFSS-DS questionnaire was given to both the parent and child patient, as shown in Table 1. Parents were requested to complete the form in the reception area and children filled the form in operatory room where dental assistants were available to assist if needed. Once completed, both questionnaires were returned and parents were allowed to join their children in the operatory room, where the planned dental treatment began and the Frankl score was given to the child, as shown in Table 2.

**Table 1:** **Table 1:** Children’s fear survey schedule—dental subscale questionnaire

1.		Dentists		1		2		3		4		5	
2.		Doctors		1		2		3		4		5	
3.		Injections		1		2		3		4		5	
4.		Having somebody examine the mouth		1		2		3		4		5	
5.		Having to open your mouth		1		2		3		4		5	
6.		Having a stranger touch you		1		2		3		4		5	
7.		Having somebody look at you		1		2		3		4		5	
8.		The dentist drilling		1		2		3		4		5	
9.		The sight of dentist drilling		1		2		3		4		5	
10.		The noise of dentist drilling		1		2		3		4		5	
11.		Having someone put instruments in your mouth		^1^		2		3		4		5	
12.		Choking		1		2		3		4		5	
13.		Having to go to the hospital		1		2		3		4		5	
14.		People in white uniforms		1		2		3		4		5	
15.		Having the nurse clean your teeth		1		2		3		4		5	
*The anxiety is marked in 5-point anxiety scale*													
1.		Not afraid at all											
2.		Very less											
3.		Moderate fear											
4.		Pretty much afraid											
5.		Very much afraid											

**Table 2:** **Table 2:** Frankl scale 1962

*Rating*			
F1		Definitely negative (—)	
F2		Negative (-)	
F3		Positive (+)	
F4		Definitely positive (++)	

### Statistical Methods

Data were entered into Microsoft Excel spreadsheet where it was summarized using tables and graphs. The data were analyzed by Statistical Package for the Social Sciences (21.0 version). Data were not normally distributed as tested using the Shapiro-Wilk W-test (p-value <0.05). Chi-square test was used for categorical variables.

Therefore, analyses were performed using the non-parametric tests, i.e., Mann-Whitney U-test. Level of statistical significance was set at p-value <0.05.

## RESULTS

**Graph 1:**
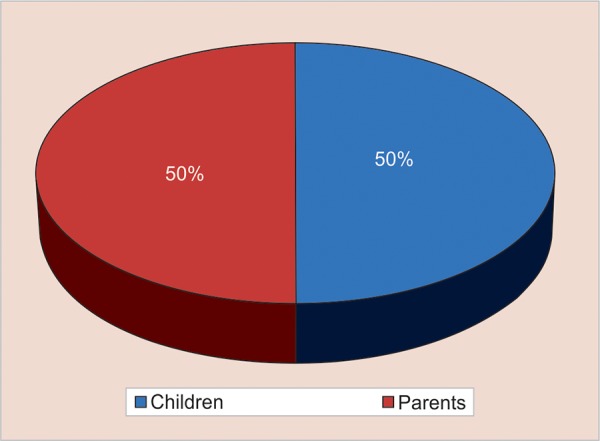
Distribution of subjects among two groups

Collected data included 94 child-parent combinations for survey. The children in this study were between 6 and 10 years old, with equal number of child-parent distribution as shown in Graph 1.

**Graph 2:**
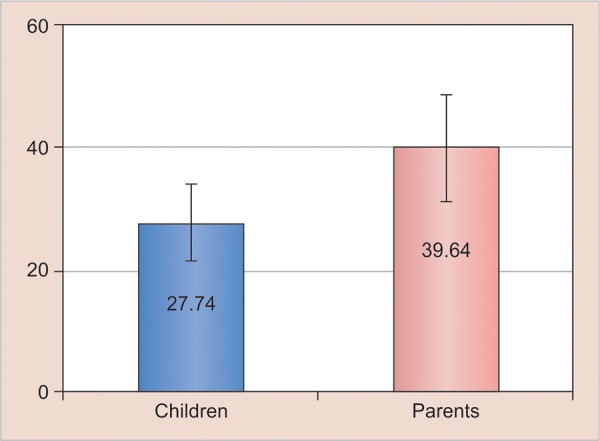
Mean of CFSS-DS among children and parents

**Table 3:** **Table 3:** Descriptive of mean and standard deviation (SD) of CFSS-DS among children and parents

		*Mean*		*SD*		*Median*		*Maximum*		*Minimum*	
Children		27.74		6.45		27		40		15	
Parents		39.64		8.64		40		75		15	

### Parents’ Ability to assess Their Child’s Dental Fear

Based on parental assessments, dental anxiety was present in 39.64% of children, whereas according to children self-report, children’s own anxiety was found to be 27.74, as shown in Table 3. This implies that parent had more anxiety for their children as compared with children’s own assessment for fear. The comparison of CFSS-DS scores among children and parents was done using Mann-Whitney U-test. It was found to be significant with parents reporting higher CFSS-DS scores as compared with children (Graph 2).

**Graph 3:**
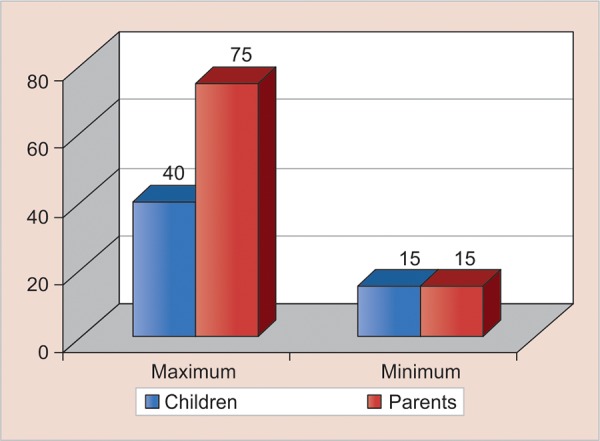
Score obtained by children and parent for their child

Maximum score obtained by parent in this study was 75 out of 75, whereas by children was 40 and minimum score obtained by parent and children was same, i.e., 15. Graph 3 signifies that parent score for their child was more as compared with child’s own dental fear score.

### The CFSS-DS Scores predicting Frankl Score

**Graph 4:**
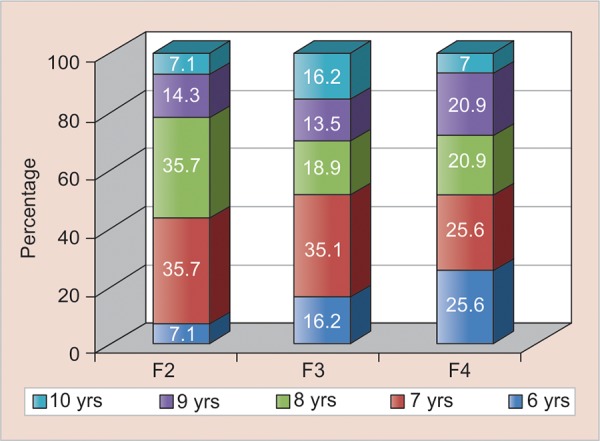
Distribution of Frankl score among children based on age

In this study, we were interested if CFSS-DS scores provided by the parent and/or child would be useful in predicting the child’s behavior, as measured via Frankl scores while in the dental operatory by the dentist. Based on this study, it was found that F2 (Frankl score-negative) was shown at the age of 7 and 8 years, whereas F3 (Frankl score-positive) was shown at the age of 7 years, and F4 (Frankl score-definitively positive) was shown between 6 and 7 years. Moreover, it was found that fear is not age-dependent (Graph 4). The comparison was done using Chi-square test and it was not found to be significant (p = 0.568) as shown in Table 4.

**Table 4:** **Table 4:** Distribution of Frankl score among children based on age

*Age in years*		*F2*				*F3*				*F4*			
		*N*		*%*		*N*		*%*		*N*		*%*	
6		1		7.1		6		16.2		11		25.6	
7		5		35.7		13		35.1		11		25.6	
8		5		35.7		7		18.9		9		20.9	
9		2		14.3		5		13.5		9		20.9	
10		1		7.1		6		16.2		3		7	
Total		14		100		37		100		43		100	
p-value		0.568											

## DISCUSSION

The CFSS-DS was developed for assessing the dental fear in children by Cuthbert and Melamed.^10^ It is a revised form of the Fear Survey Schedule for Children. Reliability of CFSS-DS in India was found to be 0.92, which is very much in agreement with the findings that was reported by Cuthbert and Melamed,^10^ Arapostathis et al,^11^ and Yamada et al.^12^ Thus, the scale was found to be reliable to assess fear.

This scale also bestows enlightenment on the most fear-eliciting aspects of dental treatment for children, which help in adopting the successful treatment planning. The easily understandable questions are attributed to the higher reliability of the scale. Luoto et al^13^ conducted a similar study on 11- to 16-year-old children and their parents, and concluded that there was no significant correlation. As there is a lack of data for younger age groups, we decided to assess the dental fear of 6- to 10-year-olds. Our results reflected that for this younger age group also, parents could not accurately predict the dental fear of their child, as shown in Table 4.

Krikken et al^14^ conducted a study in children of age group 7 to 11 years, with a different study design; this study included 326 children in a classroom setting and parental surveys were completed at home and mailed back to the patient’s schools. They reported that parents tend to overevaluate their child’s dental fear.

In our study, the mean dental anxiety score reported by the children was 27.74, while the parents’ assessment of their children was 39.64, while in a study by Krikken et al^14^ the scores were 21.15 (for children) and 23.26 (for parents). In another study, Klein et al^15^ reported that mean dental anxiety score by children was 30.30 and by parents was 33.24, which shows relatively similar result as shown by this study. In the present study, parents overestimate their child’s dental fear, which is similar to Nigerian mothers who also overestimated their children’s DFA.^16^

It was observed that younger children had lower dental fear. This could be attributed to the fact that younger children had less dental experiences than preadolescent and adolescent groups. This study reflected that the CFSS-DS helps us to predict the level and nature of a child’s dental fear adequately, but it should not be used to predict the child’s behavior during treatment.

Limitation of this study was that during this trial we met younger children who were not able to read the questions themselves, so in order to clarify the questions they were assisted by the dental official which could have impacted on a few answers.

## CONCLUSION

This study concluded that the anxiety score that was reported by children was a better anticipator of Frankl behavior score than the parental anxiety score as parents were unable to judge their children’s dental fear, as assessed by the CFSS-DS. Parents reported a higher anxiety score for their children. Parent assessment of their child’s dental fear may vary in accordance with their dental experiences.
